# Supplementary material for: Independent COL5A1 Variants in Cats with Ehlers-Danlos Syndrome
Source: Genes (Basel). 2022 Apr 29;13(5):797. doi: 10.3390/genes13050797 (PMC9140822; doi:10.3390/genes13050797)
Supplement: Supplementary file 1 [file genes-13-00797-s001.zip › Figure_S1.pdf]

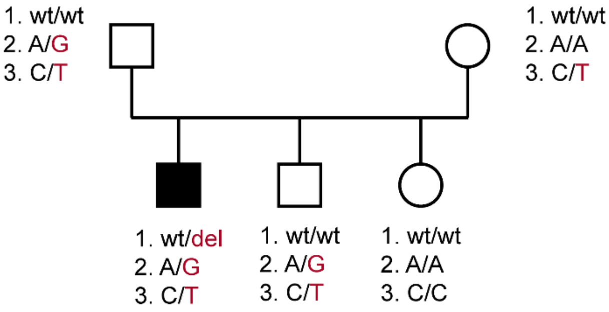


1. *COL5A1*, XM_023242950.1:c.3066del, XP_023098718.1:p.(Gly1023Valfs*50), ChrD4:93,215,490del (felCat9.0)
2. *TNXB*, XM_023254711.1:c.7084A>G, XP_023110479.1:p.(Arg2362Gly), ChrB2:32,982,832T>C (felCat9.0)
3. *TNXB*, XM_023254711.1:c.12047C>T, XP_023110479.1:p.(Ala4016Val), ChrB2:32,964,899G>A (felCat9.0)

**Figure S1.** Pedigree with genotypes from the family of case no. 4. The genotypes for all three identified protein-changing variants in EDS candidate genes from variant filtering are indicated. The correct variant designation for each variant is given. The *COL5A1* single base deletion occurred *de novo* in the affected cat and is absent from both parents. Two heterozygous variants in *TNXB* in case no. 4 were likely inherited as one haplotype from the father and therefore not likely to represent a state of compound heterozygosity.
